# 5,6-Dimethyl­pyrazine-2,3-dicarb­oxy­lic acid

**DOI:** 10.1107/S1600536811052366

**Published:** 2011-12-14

**Authors:** Fu-Hong Liu

**Affiliations:** a Basis Department, Jilin Business and Technology College, Hao Yue Road No. 1606, Changchun, Jilin, People’s Republic of China

## Abstract

The asymmetric unit of the title compound, C_8_H_8_N_2_O_4_, consists of one complete mol­ecule and a second mol­ecule generated by the application of twofold axis. The mean planes of the two carboxyl groups attached to the pyrazine ring at neighboring positions are twisted by 10.8 (1) and 87.9 (1)° in the complete molecule and 43.0 (1)° in the symmetry-generated molecule. The crystal packing features O—H⋯N hydrogen bonds, which link the mol­ecules into layers along [101].

## Related literature

For the synthesis of the title compound, see Tsuda & Fujishima (1981 ▶). For the structure of the hydrate of the title compound, see Vishweshwar *et al.* (2001 ▶, 2004 ▶). For a related compound containing pyrazine-2,3-dicarb­oxy­lic acid, see: Alborés & Rentschler (2009 ▶).
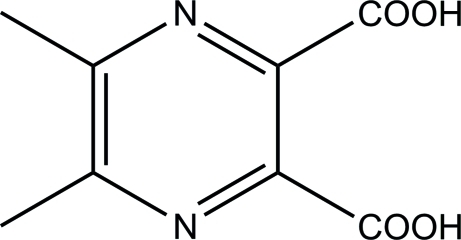


## Experimental

### 

#### Crystal data

C_8_H_8_N_2_O_4_
*M* *_r_* = 196.16
Monoclinic, 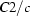

*a* = 15.873 (3) Å
*b* = 14.057 (3) Å
*c* = 11.991 (2) Å
β = 109.21 (3)°
*V* = 2526.6 (9) Å^3^
*Z* = 12
Mo *K*α radiation
μ = 0.13 mm^−1^
*T* = 293 K
0.30 × 0.25 × 0.20 mm

#### Data collection

Rigaku SCX-mini diffractometer
10832 measured reflections
2230 independent reflections
1937 reflections with *I* > 2σ(*I*)
*R*_int_ = 0.043

#### Refinement

*R*[*F* ^2^ > 2σ(*F* ^2^)] = 0.049
*wR*(*F* ^2^) = 0.133
*S* = 1.07
2230 reflections
196 parameters
H-atom parameters constrained
Δρ_max_ = 0.63 e Å^−3^
Δρ_min_ = −0.39 e Å^−3^

### 

Data collection: *PROCESS-AUTO* (Rigaku, 1998 ▶); cell refinement: *PROCESS-AUTO*; data reduction: *CrystalStructure* (Rigaku/MSC, 2002 ▶); program(s) used to solve structure: *SHELXS97* (Sheldrick, 2008 ▶); program(s) used to refine structure: *SHELXL97* (Sheldrick, 2008 ▶); molecular graphics: *ORTEP-3 for Windows* (Farrugia, 1997 ▶); software used to prepare material for publication: *publCIF* (Westrip, 2010 ▶).

## Supplementary Material

Crystal structure: contains datablock(s) I, global. DOI: 10.1107/S1600536811052366/jj2111sup1.cif

Structure factors: contains datablock(s) I. DOI: 10.1107/S1600536811052366/jj2111Isup2.hkl

Supplementary material file. DOI: 10.1107/S1600536811052366/jj2111Isup3.cml

Additional supplementary materials:  crystallographic information; 3D view; checkCIF report

## Figures and Tables

**Table 1:** Hydrogen-bond geometry (Å, °)

*D*—H⋯*A*	*D*—H	H⋯*A*	*D*⋯*A*	*D*—H⋯*A*
O1—H1⋯N2^i^	0.82 (1)	2.04	2.845 (2)	167
O3—H3⋯N3^ii^	0.82 (1)	2.00	2.803 (2)	165
O5—H5⋯N1^iii^	0.82 (1)	2.06	2.874 (2)	169

Symmetry codes: (i) 
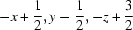
; (ii) 
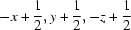
; (iii) 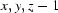.

**Fig. 1.**
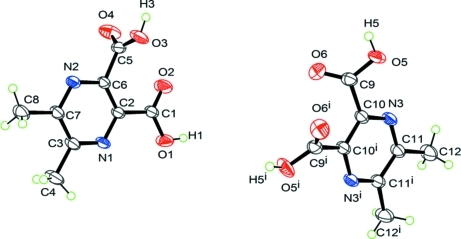
Molecular structure of (I), showing the atom-numbering scheme. Displacement ellipsoids are drawn at the 50% probability level.

**Fig. 2.**
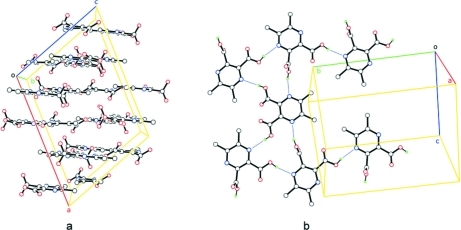
Packing view of (I). (a) View of layers in (I) along the b axis. (b) View of the title compound along the a axis. Dashed lines indicate O—H···N hydrogen bonds. H atoms not involved in hydrogen bonds have been deleted for clarity.

## supplementary crystallographic information

### Comment

2,3-dicarboxypyrazine-based ligands are well suited for the building of large clusters.  With two carboxyl groups available for binding and a non-binding site opposite, selective interactions with metal ions are common.  For example, a Co36 cluster using a pyrazine-2,3-dicarboxylic acid ligand similar to the title compound has been reported (Alborés & Rentschler, 2009) Similarly, the crystal structure of the title compound containing one water molecule in the unit cell has also been reported (Vishweshwar *et al.*, 2001; Vishweshwar *et al.*, 2004),  In view of the importance of compounds containing this ligand we report herin the crystal structure of the title compound, (I).

The asymmetric unit of the title compound, C_8_H_8_N_2_O_4_, consists of one molecule and a second complete molecule generated by the application of a centre of inversion (Fig. 1).  For each molecule, the mean planes of the two carboxyl groups attached to the pyrazine ring at neighboring positionsare twisted by 10.8 (1)° and 87.9 (3)°. Crystal packing is stabilized by O—H···N hydrogen bonds which link the molecules into layers along [101] (Fig. 2).

### Experimental

The compound was synthesized by a reported reaction (Tsuda & Fujishima, 1981) and crystallized by a solvent-thermal reaction as follows: 196.16 mg (1 mmol) C_8_H_8_N_2_O_4_ and 10 ml*N*, *N*-dimethylformamide (DMF) was added to a 20 ml Teflon vessel. The vessel was sealed and placed inside a stainless steel autoclave, which was kept at 130°C for 72 h. Crystals suitable for single-crystal analysis were formed upon standing.

### Refinement

H atoms bonded to O atom were located in a Fourier difference map and refined with distance restraints of O—H = 0.820 Å, and with *U*_iso_(H) = 1.5Ueq(O). The remaining H atoms were positioned geometrically and refined using the riding model, with C—H = 0.960 Å and with *U*_iso_(H) = 1.5 times *U*_eq_(C).

### Crystal data

**Table:**

C_8_H_8_N_2_O_4_	*F*(000) = 1224
*M**_r_* = 196.16	*D*_x_ = 1.547 Mg m^−^^3^
Monoclinic, *C*2/*c*	Mo *K*α radiation, λ = 0.71073 Å
Hall symbol: -C 2yc	Cell parameters from 10631 reflections
*a* = 15.873 (3) Å	θ = 3.4–27.8°
*b* = 14.057 (3) Å	µ = 0.13 mm^−^^1^
*c* = 11.991 (2) Å	*T* = 293 K
β = 109.21 (3)°	Block, colourless
*V* = 2526.6 (9) Å^3^	0.30 × 0.25 × 0.20 mm
*Z* = 12	

### Data collection

**Table:**

Rigaku SCX-mini diffractometer	1937 reflections with *I* > 2σ(*I*)
Radiation source: fine-focus sealed tube	*R*_int_ = 0.043
graphite	θ_max_ = 25.0°, θ_min_ = 3.4°
ω scans	*h* = −18→18
10832 measured reflections	*k* = −16→16
2230 independent reflections	*l* = −14→14

### Refinement

**Table:**

Refinement on *F*^2^	Primary atom site location: structure-invariant direct methods
Least-squares matrix: full	Secondary atom site location: difference Fourier map
*R*[*F*^2^ > 2σ(*F*^2^)] = 0.049	Hydrogen site location: inferred from neighbouring sites
*wR*(*F*^2^) = 0.133	H-atom parameters constrained
*S* = 1.07	*w* = 1/[σ^2^(*F*_o_^2^) + (0.0617*P*)^2^ + 3.0039*P*] where *P* = (*F*_o_^2^ + 2*F*_c_^2^)/3
2230 reflections	(Δ/σ)_max_ < 0.001
196 parameters	Δρ_max_ = 0.63 e Å^−^^3^
0 restraints	Δρ_min_ = −0.39 e Å^−^^3^

### Special details

**Table:**

Geometry. All e.s.d.'s (except the e.s.d. in the dihedral angle between two l.s. planes) are estimated using the full covariance matrix. The cell e.s.d.'s are taken into account individually in the estimation of e.s.d.'s in distances, angles and torsion angles; correlations between e.s.d.'s in cell parameters are only used when they are defined by crystal symmetry. An approximate (isotropic) treatment of cell e.s.d.'s is used for estimating e.s.d.'s involving l.s. planes.
Refinement. Refinement of *F*^2^ against ALL reflections. The weighted *R*-factor *wR* and goodness of fit *S* are based on *F*^2^, conventional *R*-factors *R* are based on *F*, with *F* set to zero for negative *F*^2^. The threshold expression of *F*^2^ > σ(*F*^2^) is used only for calculating *R*-factors(gt) *etc*. and is not relevant to the choice of reflections for refinement. *R*-factors based on *F*^2^ are statistically about twice as large as those based on *F*, and *R*- factors based on ALL data will be even larger.

### Fractional atomic coordinates and isotropic or equivalent isotropic displacement parameters (Å^2^)

**Table:**

	*x*	*y*	*z*	*U*_iso_*/*U*_eq_	
O1	0.28005 (14)	0.38972 (11)	0.78999 (16)	0.0595 (6)	
H1	0.2587	0.3468	0.7428	0.089*	
O2	0.17345 (15)	0.47314 (13)	0.66739 (18)	0.0745 (7)	
O3	0.20112 (11)	0.67092 (14)	0.57803 (14)	0.0482 (5)	
H3	0.1591	0.6847	0.5190	0.072*	
O4	0.09881 (12)	0.67291 (15)	0.66709 (16)	0.0567 (5)	
O5	0.40546 (13)	0.35937 (12)	0.02848 (13)	0.0474 (5)	
H5	0.3815	0.4101	0.0026	0.071*	
O6	0.40159 (11)	0.41517 (11)	0.20060 (15)	0.0439 (4)	
N1	0.34098 (12)	0.54156 (12)	0.92729 (14)	0.0318 (4)	
N2	0.28289 (12)	0.72021 (12)	0.84036 (15)	0.0325 (4)	
N3	0.43566 (12)	0.18493 (12)	0.14017 (14)	0.0311 (4)	
C1	0.23855 (16)	0.46795 (15)	0.75119 (19)	0.0362 (5)	
C2	0.27656 (14)	0.55308 (14)	0.82342 (17)	0.0291 (5)	
C3	0.37616 (14)	0.61868 (15)	0.98780 (18)	0.0325 (5)	
C4	0.44770 (18)	0.60708 (18)	1.1037 (2)	0.0491 (7)	
H4A	0.4231	0.6160	1.1662	0.074*	
H4B	0.4936	0.6534	1.1110	0.074*	
H4C	0.4726	0.5444	1.1090	0.074*	
C5	0.17291 (15)	0.66150 (14)	0.66717 (19)	0.0324 (5)	
C6	0.24752 (13)	0.64193 (14)	0.78045 (17)	0.0276 (5)	
C7	0.34688 (14)	0.70945 (15)	0.94401 (18)	0.0315 (5)	
C8	0.38542 (18)	0.79617 (17)	1.0119 (2)	0.0489 (7)	
H8A	0.4458	0.8040	1.0132	0.073*	
H8B	0.3846	0.7900	1.0913	0.073*	
H8C	0.3508	0.8506	0.9752	0.073*	
C9	0.42126 (14)	0.35590 (15)	0.14246 (18)	0.0316 (5)	
C10	0.46645 (14)	0.26613 (14)	0.19620 (17)	0.0286 (5)	
C11	0.46841 (15)	0.10400 (15)	0.19300 (18)	0.0315 (5)	
C12	0.43794 (18)	0.01403 (16)	0.1277 (2)	0.0462 (6)	
H12A	0.3870	0.0264	0.0593	0.069*	
H12B	0.4220	−0.0306	0.1780	0.069*	
H12C	0.4851	−0.0120	0.1036	0.069*	

### Atomic displacement parameters (Å^2^)

**Table:**

	*U*^11^	*U*^22^	*U*^33^	*U*^12^	*U*^13^	*U*^23^
O1	0.0717 (13)	0.0232 (8)	0.0508 (11)	0.0069 (8)	−0.0244 (9)	−0.0016 (7)
O2	0.0871 (15)	0.0368 (10)	0.0546 (12)	0.0090 (10)	−0.0375 (11)	−0.0110 (9)
O3	0.0448 (10)	0.0646 (12)	0.0236 (8)	0.0094 (9)	−0.0042 (7)	0.0075 (8)
O4	0.0354 (10)	0.0753 (14)	0.0467 (11)	0.0054 (9)	−0.0037 (8)	0.0138 (9)
O5	0.0688 (12)	0.0361 (9)	0.0240 (8)	0.0145 (9)	−0.0028 (8)	0.0065 (7)
O6	0.0525 (11)	0.0343 (9)	0.0447 (10)	0.0100 (8)	0.0157 (8)	0.0008 (7)
N1	0.0353 (10)	0.0277 (9)	0.0221 (9)	−0.0014 (8)	−0.0044 (8)	0.0045 (7)
N2	0.0346 (10)	0.0269 (9)	0.0257 (9)	−0.0005 (8)	−0.0040 (8)	0.0010 (7)
N3	0.0375 (10)	0.0278 (9)	0.0201 (9)	−0.0013 (8)	−0.0014 (7)	−0.0005 (7)
C1	0.0447 (13)	0.0259 (11)	0.0258 (11)	−0.0001 (10)	−0.0049 (10)	0.0017 (9)
C2	0.0313 (11)	0.0263 (11)	0.0223 (10)	−0.0008 (9)	−0.0011 (8)	0.0021 (8)
C3	0.0344 (12)	0.0310 (11)	0.0232 (11)	−0.0061 (9)	−0.0027 (9)	0.0032 (8)
C4	0.0528 (16)	0.0414 (13)	0.0319 (13)	−0.0110 (12)	−0.0148 (11)	0.0073 (10)
C5	0.0341 (13)	0.0226 (11)	0.0295 (12)	0.0000 (9)	−0.0042 (9)	0.0024 (8)
C6	0.0291 (11)	0.0241 (10)	0.0226 (10)	−0.0021 (9)	−0.0011 (8)	−0.0004 (8)
C7	0.0340 (12)	0.0298 (11)	0.0229 (11)	−0.0052 (9)	−0.0012 (9)	0.0002 (8)
C8	0.0577 (16)	0.0324 (13)	0.0369 (14)	−0.0090 (11)	−0.0111 (12)	−0.0027 (10)
C9	0.0312 (11)	0.0287 (11)	0.0275 (11)	−0.0012 (9)	−0.0006 (9)	0.0019 (9)
C10	0.0339 (11)	0.0263 (11)	0.0210 (10)	−0.0003 (9)	0.0026 (8)	−0.0011 (8)
C11	0.0392 (12)	0.0260 (11)	0.0239 (11)	−0.0021 (9)	0.0027 (9)	−0.0010 (8)
C12	0.0618 (16)	0.0295 (12)	0.0338 (13)	−0.0040 (11)	−0.0025 (11)	−0.0054 (10)

### Geometric parameters (Å, °)

**Table:**

O1—C1	1.288 (3)	C3—C7	1.400 (3)
O1—H1	0.8200	C3—C4	1.487 (3)
O2—C1	1.183 (3)	C4—H4A	0.9600
O3—C5	1.294 (3)	C4—H4B	0.9600
O3—H3	0.8200	C4—H4C	0.9600
O4—C5	1.187 (3)	C5—C6	1.505 (3)
O5—C9	1.307 (3)	C7—C8	1.482 (3)
O5—H5	0.8200	C8—H8A	0.9600
O6—C9	1.192 (3)	C8—H8B	0.9600
N1—C3	1.322 (3)	C8—H8C	0.9600
N1—C2	1.336 (3)	C9—C10	1.489 (3)
N2—C7	1.330 (3)	C10—C10^i^	1.377 (4)
N2—C6	1.333 (3)	C11—C11^i^	1.405 (4)
N3—C11	1.323 (3)	C11—C12	1.482 (3)
N3—C10	1.333 (3)	C12—H12A	0.9600
C1—C2	1.484 (3)	C12—H12B	0.9600
C2—C6	1.372 (3)	C12—H12C	0.9600
			
C1—O1—H1	109.5	C2—C6—C5	124.95 (18)
C5—O3—H3	109.5	N2—C7—C3	120.76 (18)
C9—O5—H5	109.5	N2—C7—C8	118.06 (19)
C3—N1—C2	117.91 (17)	C3—C7—C8	121.17 (19)
C7—N2—C6	117.82 (18)	C7—C8—H8A	109.5
C11—N3—C10	118.29 (17)	C7—C8—H8B	109.5
O2—C1—O1	124.0 (2)	H8A—C8—H8B	109.5
O2—C1—C2	121.3 (2)	C7—C8—H8C	109.5
O1—C1—C2	114.60 (18)	H8A—C8—H8C	109.5
N1—C2—C6	121.33 (18)	H8B—C8—H8C	109.5
N1—C2—C1	119.07 (18)	O6—C9—O5	126.0 (2)
C6—C2—C1	119.54 (18)	O6—C9—C10	121.38 (19)
N1—C3—C7	120.89 (18)	O5—C9—C10	112.56 (19)
N1—C3—C4	118.57 (19)	N3—C10—C10^i^	120.98 (11)
C7—C3—C4	120.54 (19)	N3—C10—C9	117.57 (18)
C3—C4—H4A	109.5	C10^i^—C10—C9	121.30 (11)
C3—C4—H4B	109.5	N3—C11—C11^i^	120.57 (11)
H4A—C4—H4B	109.5	N3—C11—C12	118.19 (19)
C3—C4—H4C	109.5	C11^i^—C11—C12	121.23 (13)
H4A—C4—H4C	109.5	C11—C12—H12A	109.5
H4B—C4—H4C	109.5	C11—C12—H12B	109.5
O4—C5—O3	126.7 (2)	H12A—C12—H12B	109.5
O4—C5—C6	120.7 (2)	C11—C12—H12C	109.5
O3—C5—C6	112.4 (2)	H12A—C12—H12C	109.5
N2—C6—C2	121.28 (18)	H12B—C12—H12C	109.5
N2—C6—C5	113.73 (17)		
			
C3—N1—C2—C6	0.0 (3)	O4—C5—C6—C2	93.3 (3)
C3—N1—C2—C1	177.4 (2)	O3—C5—C6—C2	−91.5 (3)
O2—C1—C2—N1	169.2 (3)	C6—N2—C7—C3	0.4 (3)
O1—C1—C2—N1	−8.3 (3)	C6—N2—C7—C8	−179.0 (2)
O2—C1—C2—C6	−13.3 (4)	N1—C3—C7—N2	0.0 (3)
O1—C1—C2—C6	169.1 (2)	C4—C3—C7—N2	−180.0 (2)
C2—N1—C3—C7	−0.2 (3)	N1—C3—C7—C8	179.4 (2)
C2—N1—C3—C4	179.8 (2)	C4—C3—C7—C8	−0.6 (4)
C7—N2—C6—C2	−0.6 (3)	C11—N3—C10—C10^i^	3.0 (4)
C7—N2—C6—C5	177.3 (2)	C11—N3—C10—C9	−172.7 (2)
N1—C2—C6—N2	0.5 (3)	O6—C9—C10—N3	134.4 (2)
C1—C2—C6—N2	−177.0 (2)	O5—C9—C10—N3	−44.4 (3)
N1—C2—C6—C5	−177.3 (2)	O6—C9—C10—C10^i^	−41.3 (4)
C1—C2—C6—C5	5.3 (3)	O5—C9—C10—C10^i^	140.0 (3)
O4—C5—C6—N2	−84.5 (3)	C10—N3—C11—C11^i^	2.3 (4)
O3—C5—C6—N2	90.6 (2)	C10—N3—C11—C12	−176.5 (2)

Symmetry codes: (i) −*x*+1, *y*, −*z*+1/2.

### Hydrogen-bond geometry (Å, °)

**Table:**

*D*—H···*A*	*D*—H	H···*A*	*D*···*A*	*D*—H···*A*
O1—H1···N2^ii^	0.82 (1)	2.04	2.845 (2)	167.
O3—H3···N3^iii^	0.82 (1)	2.00	2.803 (2)	165.
O5—H5···N1^iv^	0.82 (1)	2.06	2.874 (2)	169.

Symmetry codes: (ii) −*x*+1/2, *y*−1/2, −*z*+3/2; (iii) −*x*+1/2, *y*+1/2, −*z*+1/2; (iv) *x*, *y*, *z*−1.
